# Acute Air Pollutant Exposure and Cognitive Impairments in Healthy Young Adults: A Scoping Review of Human-Controlled Exposure Studies

**DOI:** 10.7759/cureus.108611

**Published:** 2026-05-10

**Authors:** Shreehari Shreedhar, Dan Li

**Affiliations:** 1 Public Health, Yasmina British Academy, Abu Dhabi, ARE; 2 Psychiatry, Massachusetts General Hospital, Boston, USA

**Keywords:** air pollution, cognitive impairment, neurodegeneration, public health, social determinants of health

## Abstract

Over the past decade, ambient air pollutant concentrations have increased, largely attributed to escalating energy consumption. Emerging evidence suggests that prolonged or repeated exposure to air pollutants may significantly accelerate the onset and progression of cognitive impairment, with studies reporting up to a 64% increase in systemic stress biomarkers and measurable cognitive deficits occurring within minutes of inhalation. Accordingly, this scoping review aimed to synthesize evidence from controlled human exposure studies investigating the relationship between acute air pollution exposure and neurological outcomes in healthy adults. To address this aim, eligible studies included literature examining exposure to airborne pollutants and assessing cognitive and neurobiological outcomes in human participants. A systematic search of PubMed was conducted using predefined MeSH terms and title/abstract keywords related to air pollution and cognition. Only full-text, English-language randomized clinical trials involving young adults were included. The study selection process was documented in accordance with the PRISMA guidelines, and methodological quality was assessed using the Joanna Briggs Institute (JBI) critical appraisal tool. Due to heterogeneity across studies, results were synthesized descriptively. Six clinical trials published between 2008 and 2024 met the inclusion criteria, with sample sizes ranging from 12 to 72 participants. Pollutants investigated included particulate matter, diesel exhaust, and elevated carbon dioxide concentrations. Across the included studies, these exposures were associated with impairments in executive function, attention, memory, and reaction time. Diesel exhaust exposure was associated with slower reaction times, while carbon dioxide inhalation increased working memory errors. Exposure to particulate matter was also associated with elevations in stress-related biomarkers. In contrast, filtered air conditions were associated with improved cognitive performance. Overall, acute exposure to air pollutants may produce measurable cognitive and neurobiological effects even in healthy adults, suggesting potential neurological vulnerability regardless of pre-existing conditions. However, the current evidence base remains limited. Future research should prioritize large-scale, ecologically valid studies to better define the mechanisms through which air pollutants disrupt neurological processes.

## Introduction and background

Air pollution is widely recognized as one of the world’s leading environmental risk factors affecting human health. Numerous studies in the medical literature have linked exposure to airborne pollutants, including particulate matter (a mixture of small particles and liquid droplets), diesel exhaust, and nitrogen oxides, to adverse respiratory and cardiovascular outcomes [1,2]. In particular, PM2.5 (particulate matters with diameters of 2.5 μm or less) is of significant concern because of its ability to bypass the body’s innate defense mechanisms and enter the bloodstream. Increasing attention has also been directed toward the neurological consequences of air pollutant exposure, including its effects on cognitive performance and brain function [3]. Epidemiological studies suggest that air pollutants may impair neurocognitive processes through several biological mechanisms, including oxidative stress, neuroinflammation, and disruption of the blood-brain barrier [4]. Oxidative stress occurs when pollutants trigger the overproduction of reactive oxygen species that overwhelm cellular antioxidant defenses, while neuroinflammation involves activation of the brain’s immune cells. Together, these mechanisms may compromise the integrity of the blood-brain barrier, a highly selective semipermeable structure that protects the brain. Disruption of this barrier may permit toxins and inflammatory mediators to enter the brain parenchyma.

Furthermore, inhaled particulate matter may reach the central nervous system either through a compromised blood-brain barrier or via axonal transport through the olfactory bulb. Once present within the brain parenchyma, these pollutants can initiate an inflammatory cascade characterized by activation of pro-inflammatory cytokines. This neuroinflammatory environment, combined with pollutant-induced oxidative stress, may contribute to mitochondrial dysfunction within neurons, potentially impairing executive function and processing speed. 

Although research investigating the health effects of air pollution has expanded in recent years, much of the existing literature has focused on the consequences of long-term exposure among aging individuals and populations with preexisting conditions. In contrast, there remains a relative lack of synthesized evidence regarding the acute effects of short-term air pollutant exposure. Limited empirical data are available on how acute exposure affects healthy young adults and whether neurobiological resilience may mask early cognitive or neurological changes. For the purposes of this review, “healthy adults” were defined as individuals between 18 and 50 years of age with no history of chronic respiratory, cardiovascular, or neurological disease.

Research gap

Relatively little attention has been given to the short-term effects of acute air pollutant exposure in healthy young adults. Understanding these acute effects is important, as individuals are frequently exposed to short periods of elevated air pollutant levels, particularly in urban environments. Controlled human exposure studies provide valuable insight into these short-term effects under carefully monitored conditions; however, findings from these studies remain dispersed across multiple disciplines, including occupational medicine, environmental health, and neuropsychology. Consequently, the current evidence base lacks a comprehensive synthesis of experimental data examining how acute air pollutant exposure may influence cognitive processes.

Objectives and implications

In accordance with the PRISMA-ScR framework, the primary objective of this review is to map the extent, range, and nature of evidence from controlled human exposure studies investigating the relationship between acute air pollutant exposure and cognitive and neurobiological outcomes in healthy adults. Additionally, the review aims to examine the underlying biological mechanisms linking acute air pollutant exposure to these outcomes.

By identifying and consolidating the current evidence, this review seeks to establish a more precise baseline for understanding neurobiological resilience in non-vulnerable populations. This is particularly important because most public health guidelines are derived from chronic exposure data obtained from high-risk cohorts. Strengthening the evidence base regarding the potential short-term neurological effects of acute air pollutant exposure is therefore critical. Improved understanding of these acute effects is essential for developing comprehensive risk assessments and informing public health strategies that promote health equity within increasingly urbanized environments.

## Review

Methods

Study Design

This study was conducted as a scoping review to map the existing data and clinical evidence examining the effects of air pollution on cognitive and neurobiological outcomes in young adults. A descriptive synthesis was employed rather than a meta-analysis due to the high degree of heterogeneity among studies, with respect to pollutant types, exposure durations, and the wide range of neuropsychological assessment tools utilized. This heterogeneity precluded the calculation of a pooled effect size. The review methodology was conducted in accordance with the PRISMA-ScR guidelines for scoping reviews [5].

Search Strategy

A systematic literature search was conducted using PubMed to identify relevant studies. The search strategy utilized Boolean operators (OR/AND) to combine MeSH terms and title/abstract (TiAb) keywords. MeSH terms related to air pollution exposure included “Air Pollution”, “Particulate Matter”, “Vehicle Emissions”, “Nitrogen Dioxide”, and “Carbon Monoxide”, along with related TiAb keywords such as “traffic-related air pollution”, “PM2.5”, and “PM10”. Cognitive outcome terms included MeSH terms and keywords such as “Cognition”, “Cognitive Dysfunction”, “Executive Function”, “Memory”, “Attention”, “Processing Speed”, and “Neuropsychological Tests”. These terms were first searched individually using the OR operator and subsequently combined using the AND operator. Database filters were applied in accordance with the predefined inclusion and exclusion criteria to refine the search results and identify relevant studies.

Study Selection and Screening

**Figure 1:**
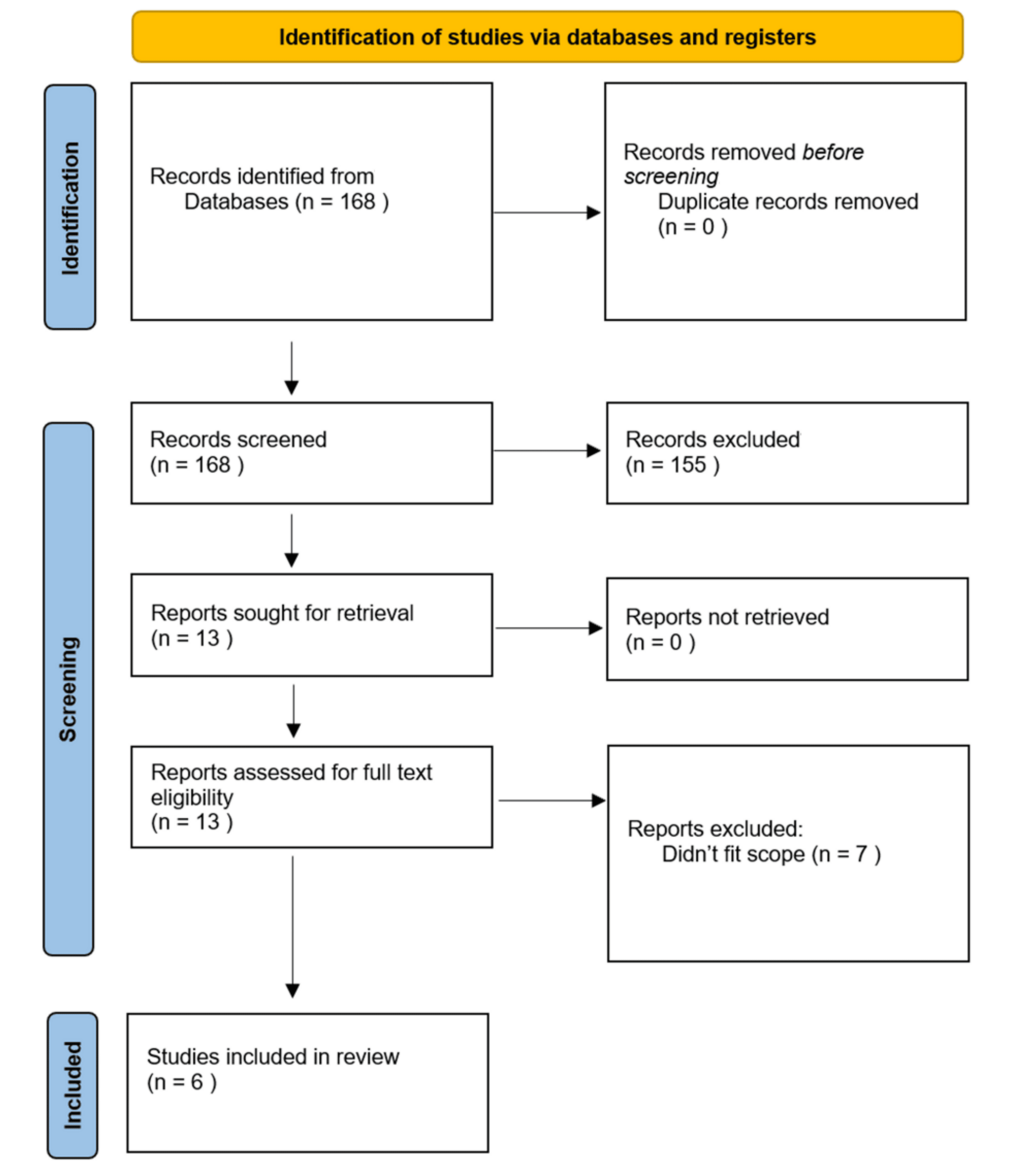
Flow chart of the study selection process.

The study selection process was conducted in two stages. The initial stage involved title and abstract screening of all studies identified through the database search. In the second stage, full-text review of potentially relevant articles was performed and evaluated against the predefined inclusion and exclusion criteria. Additionally, a second independent reviewer conducted title and abstract screening to enhance selection reliability. The study selection process was documented using a PRISMA flow diagram illustrating the identification, screening, eligibility assessment, and inclusion of studies (Figure 1).

Inclusion and Exclusion Criteria

Studies were included if they investigated the relationship between air pollution exposure and cognitive or neurobiological outcomes in humans. No geographical or population-based restrictions were applied. The conceptual focus of the review centered on air pollution exposure, cognitive impairment, and young adults. Eligible study designs included randomized clinical trials that assessed cognitive performance or neurobiological markers following exposure to air pollutants. Only studies involving human participants were considered. Articles were restricted to English-language publications published between 2008 and 2026 and available as full free text. Studies were excluded if they did not involve human participants, were not randomized clinical trials, did not evaluate cognitive outcomes or neurobiological indicators related to air pollutant exposure, were not written in English, or were unavailable as full free text. 

Risk-of-Bias Assessment

**Figure 2:**
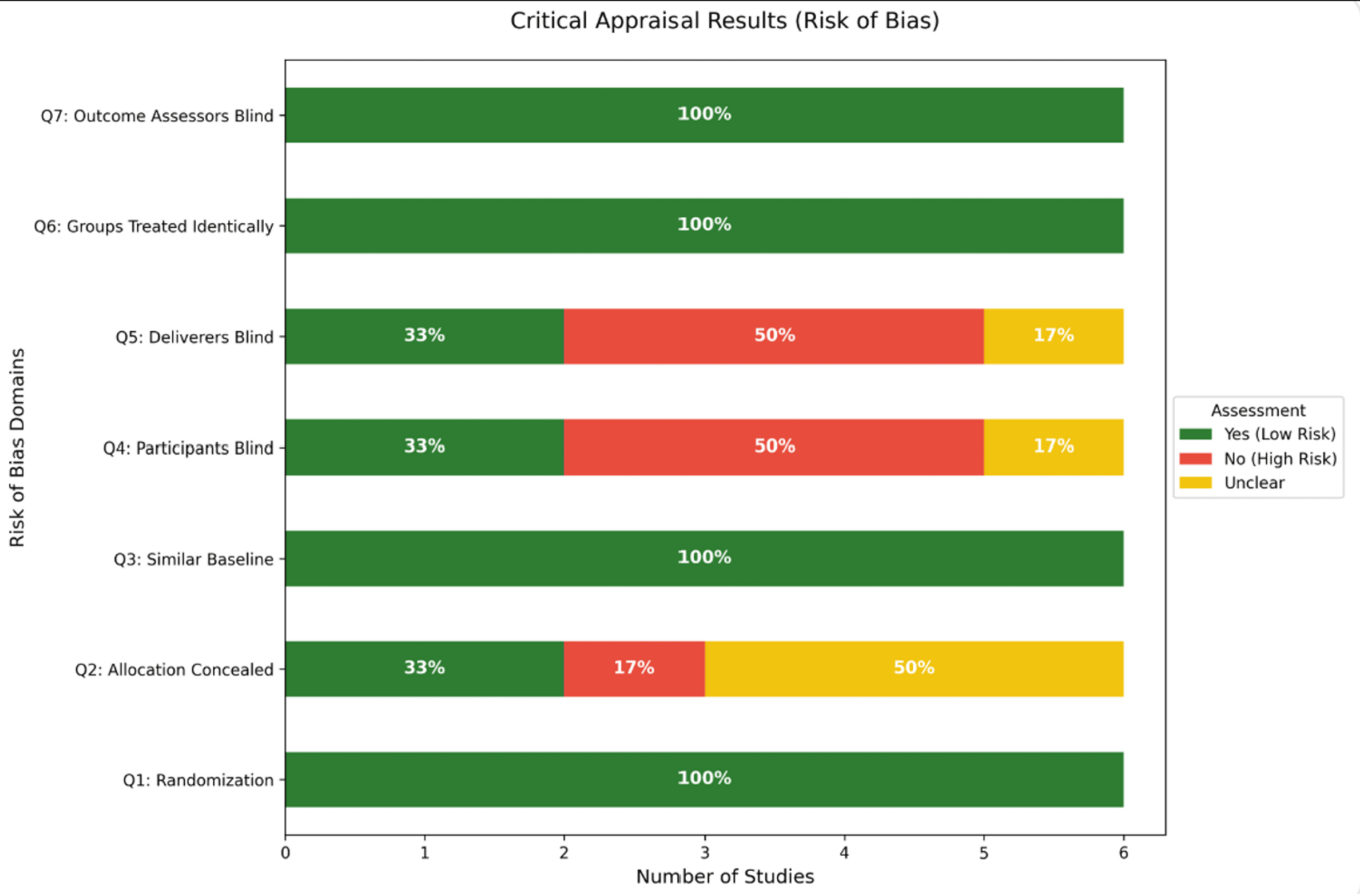
Results of the risk-of-bias assessment of included studies.

Risk of bias within included studies was assessed using the JBI critical appraisal tool for randomized controlled trials [6]. Assessment focused on study design, exposure criteria, participant selection, and measured outcomes (Figure 2). Greater validity in the included studies yielded stronger associations between air pollutants and various cognitive impairments. As illustrated in Figure 2, all included studies generally demonstrated high methodological quality, with all six trials meeting the criteria of randomized assignment and blinded outcome assessment. High validity among included studies suggests a tangible association between air pollutants and cognitive impairments.

Results

A total of six clinical trial studies published between 2008 and 2024 met the inclusion criteria. All studies employed controlled human exposure designs, predominantly randomized controlled trials, enabling comparison across exposure conditions. Sample sizes ranged from 12 to 72 participants, with most studies including healthy young adults aged approximately 26 to 36 years. All studies were conducted in urban settings worldwide, primarily in upper-middle-income countries, including China, Canada, the United Kingdom, and the Netherlands. The majority of the included clinical studies were conducted in Canada (Tables 1, 2).

**Table 1:** Study characteristics and participant demographics of included studies.

Author (year)	Country and region	Study design	Sample size	Age profile	Gender distribution
Zhou et al. (2024) [7]	Beijing, China (East Asia)	Randomized, single-blind, two-way crossover intervention study	60	Range: 18-40 years (young adults), categorized into 18-30 (52.5%) and 31-40 (47.5%)	Female: 28 participants (70%); male: 12 participants (30%)
Curran et al. (2025) [8]	Canada	Double-blinded, randomized controlled crossover study	28	Range: 19-49 years; mean age: 28 years	Female: 12 (43%); male: 16 (57%)
Savulich et al. (2019) [9]	United Kingdom	Single-blind, placebo-controlled, randomized, crossover, within-subjects study	72	Mean: 29.25 years	Experiment 1: female, 22 (50%). Experiment 2: female, 14 (52%)
Liu et al. (2017) [10]	Toronto, Canada	Single-blind, randomized controlled crossover trial	50	Mean age: 28 ± 9 years	Female: 29 (53%); male: 26 (47%)
Colasanti et al. (2008) [11]	Maastricht, Netherlands	Double-blind, randomized, controlled crossover design	64	35.8 ± 15.9 years; females: 31.1 ± 14.4 years	Male: 33 (51.5%); female: 31 (48.5%)
Hosseini et al. (2016) [12]	Vancouver, Canada	Double-blinded, randomized crossover study with a four-week washout	12	Mean: 29 ± 7 years	Female: 58%; male: 42%

**Table 2:** Exposure duration, cognitive domains, and assessment tools used in included studies.

Author (year)	Inclusion/exclusion criteria	Assessment method	Exposure duration	Cognitive domain	Assessment tool
Zhou et al. (2024) [7]	Non-smoking, healthy, no prescription/antiarrhythmic drugs, no psychiatric/learning disorders, no COVID-19 symptoms	Real-time environmental sensors	5 hours of exposure prior to testing	Memory, attention, and executive function	CogniFit® General Cognitive Assessment Battery
Curran et al. (2025) [8]	Healthy, non-smoker, English proficiency, no color-blindness, not pregnant/breastfeeding, no metal implants (for fMRI), no claustrophobia	Controlled exposure booth using a 5.5-kW diesel engine	Short-term (120 minutes per condition)	Attention, spatial working memory, strategy used, executive function, and processing speed	Cambridge Neuropsychological Test Automated Battery (CANTAB®)
Savulich et al. (2019) [9]	No psychiatric/neurological conditions; non-smokers; no first-degree relatives with panic disorder; no regular medication	Inhalation via nasal-oral mask from a 100 L Douglas bag	Acute (maximum of 20 minutes)	Cognitive flexibility (set-shifting), emotional processing (affective bias), and spatial working memory	CANTAB®
Liu et al. (2017) [10]	Healthy non-smokers, no cardiovascular/respiratory disease, no diabetes, no pregnancy/breastfeeding	Controlled exposure chamber using Harvard Ambient Particle Concentrators	Short-term (130 minutes)	Blood-brain barrier (BBB) Integrity and systemic stress response	ELISA assays for blood/urinary biomarkers
Colasanti et al. (2008) [11]	Healthy 18-65-year-olds. Exclusion: history of pulmonary/cardiovascular disease, hypertension, pregnancy, epilepsy, heavy smoking (>15 cigarettes/day), psychotropic medication, or family history of anxiety/affective disorders	Double vital capacity inhalation of four different gas mixtures via a face mask	Acute (brief double-breath inhalation); tested on four different days within one week	Subjective fear/discomfort and DSM-IV panic symptomatology	Panic Symptom List (PSL-IV): 13 items based on DSM criteria
Hosseini et al. (2016) [12]	Atopic (allergic) sensitization to house dust mite (HDM), birch, or grasses; FEV1 geq 63% predicted	Controlled inhalation in an exposure booth (diesel exhaust) and segmental allergen challenge (SAC) via bronchoscopy	2 hours of DE/filtered air, followed by SAC 1 hour later	Study has examined inflammatory rather than cognitive outcome	Bronchial biopsies, GMA immunohistochemistry, and morphometric analysis

Across studies, participant recruitment occurred in community, workplace, clinical research, and academic hospital settings. Funding sources were largely unreported. Most samples were balanced by sex, although two studies included a higher proportion of female participants. Socioeconomic status, when reported, was generally high, with one study indicating that 87.5% of participants held a master’s degree or higher [7].

Exposure Characteristics

All included studies assessed short-term or acute exposure to common air pollutants or toxic gas mixtures under controlled conditions. Exposure durations ranged from brief inhalation periods to up to five hours of continuous exposure. The pollutants investigated included coarse (2.5-10 μm, mean 213 μg/m³) and fine particulate matter (0.15-2.5 μm, mean 238 μg/m³), as well as diesel exhaust (300 μg/m³). These concentrations represent a substantial increase above World Health Organization global air quality guidelines, which recommend a 24-hour mean limit of 15 μg/m³ for PM2.5 [2]. Carbon dioxide (CO₂) was also investigated at concentrations ranging from 7.5% to 35%, alongside HEPA-filtered air as a control condition. Five studies used ambient or concentrated ambient particulate matter, while two studies used experimental gas mixtures. Exposure assessment methods included real-time environmental sensors, exposure chambers, Douglas bags, and diesel exhaust generation systems. The use of standardized and controlled exposure systems across studies supported strong internal validity. CO₂ exposure was included alongside ambient pollutants to model indoor ventilation-related stressors in comparison with outdoor particulate matter exposure, providing a broader assessment of acute inhalation effects on cognitive function.

Neurobiological and Cognitive Outcomes

Four studies directly evaluated cognitive performance, while the remaining two focused on neurobiological and inflammatory markers related to brain function. The cognitive domains assessed included memory, attention, executive function, strategy use, processing speed, cognitive flexibility, and emotional processing. Validated neuropsychological assessment tools were consistently used across studies, including CANTAB® and CogniFit®, with outcomes reported as continuous variables. One study reported cultural adaptation of cognitive testing through translation into Chinese to accommodate the study population and reduce language barriers.

The remaining studies examined biological or affective outcomes, including blood-brain barrier integrity, stress and inflammatory biomarkers, and panic and fear symptomatology. Assessment methods included enzyme-linked immunosorbent assays (ELISA), bronchial biopsies, and standardized psychological symptom scales.

Across the included studies, acute exposure to high concentrations of pollutants was consistently associated with poorer cognitive performance, whereas filtration and reduced exposure conditions were associated with improved outcomes. A workplace intervention study reported improved performance in 9 of 16 cognitive domains following HEPA filtration, with significant improvements in memory, attention, and executive function (p < 0.05) [7]. Diesel exhaust exposure was associated with slowed reaction times, including an increase of 18.2 ms in simple reaction time (p = 0.05) and 23.5 ms in five-choice reaction time compared with filtered air [8]. Acute CO₂ inhalation was significantly associated with increased shifting errors (d = 0.40) and spatial working memory errors (d = 0.63), with effects observed after ≤20 minutes of exposure [9]. Reported effects were adjusted for sex, age, time point, and baseline performance.

Short-term exposure to particulate matter and diesel exhaust was also associated with neurobiological stress responses. Concentrated coarse particulate exposure resulted in a 20% increase in urinary vanillylmandelic acid and a 64% increase in urinary cortisol (p < 0.05) [10]. CO₂ exposure was associated with increased panic and fear symptoms, with respiratory symptoms accounting for 65% of total symptom variance in the study [11].

Statistical Methodology and Confounding Adjustment

Most of the included studies used repeated-measures analysis of variance (ANOVA) and mixed-effects models, which were appropriate for crossover study designs. Adjustments for potential confounders were variably applied across studies, including age, sex, body mass index (BMI), season, time of testing, and baseline performance scores where appropriate. Interaction analyses were conducted in several studies and identified sex-related differences in cognitive outcomes, age-related effects on pollution-associated reaction times, and differential inflammatory responses under combined pollutant and allergen exposure conditions. However, reporting of confidence intervals was inconsistent across studies.

Most studies accounted for key confounders, including age, sex, BMI, and testing time. Several studies also performed subgroup analyses to explore effect modification. These analyses identified sex-specific effects on cognitive and emotional outcomes, age-related modification of pollution-associated reaction time changes, and interactions between air pollution and allergen exposure. Mediation analysis was infrequently performed; however, one study identified heart rate variability as a partial mediator of pollution-related changes in cognitive performance.

Biological Correlations and Mechanistic Indicators

Three of the included studies reported biological endpoints relevant to neurocognitive processes. Acute particulate exposure was associated with increases in systemic stress biomarkers, including a 20% increase in urinary vanillylmandelic acid and a 64% increase in cortisol, along with activation of sympathetic pathways [10]. In diesel exhaust-allergen models, inflammatory markers, including CD4+ T cells and Th2 cytokines, were significantly elevated compared with control conditions, indicating enhanced immune activation under combined exposures [12]. CO₂ dose-response data demonstrated a progressive increase in panic-related symptoms with increasing concentrations, with respiratory symptoms accounting for a substantial proportion of variance (65%), suggesting physiological sensitivity to hypercapnic conditions [11]. Overall, the included studies provide important physiological context for acute exposure effects; however, none established a direct link between biomarker changes and long-term functional brain outcomes.

Geographical and Population Study Areas

A majority of the included studies were conducted in Canada, with additional studies taking place in the United Kingdom, China, and the Netherlands. All study samples consisted of healthy adults without major chronic diseases, which limits generalizability to vulnerable populations such as older adults, children, or individuals with pre-existing neurological conditions, as these groups were either underrepresented or not included in the selected studies. Educational attainment was generally high across studies, and available socioeconomic indicators suggested that participants were predominantly from relatively affluent backgrounds.

Discussion

By synthesizing evidence from human-controlled exposure trials investigating the relationship between acute air pollution exposure and neurological outcomes in healthy adults, this review reinforces that air pollution is widely recognized as a major environmental risk factor, with growing evidence suggesting that exposure to air pollutants may affect neurological and cognitive processes [9-11]. While a substantial body of literature has focused on the long-term cognitive effects of air pollution in predominantly aging populations, short-term cognitive effects in healthy young adults remain comparatively underexplored. This scoping review synthesizes evidence from six controlled human exposure trials examining the intersection of acute air pollutant exposure, cognitive outcomes, and neurobiological responses [7,9-12]. Across the included studies, short-term exposure to air pollutants, including diesel exhaust, fine and coarse particulate matter (PM2.5 and PM2.5-10), and elevated carbon dioxide concentrations, was associated with statistically significant impairments in cognitive performance, particularly in domains related to executive function, memory, attention, and reaction time [7-9]. Furthermore, several studies identified biological responses to pollutant exposure, including elevated stress and inflammatory biomarkers, suggesting the potential presence of mechanistic pathways linking environmental exposure to neurocognitive effects [10,12].

Key Findings in Existing Literature

A notable finding across all included studies was the consistent detection of changes in cognitive performance following relatively short exposure to air pollutants, in some cases occurring within minutes of initial exposure. This suggests that certain cognitive processes may be sensitive to acute environmental conditions even in healthy young adults. The observed slowing of reaction times following diesel exhaust exposure (up to 23.5 ms) and increased working memory errors during CO₂ inhalation experiments (d = 0.63) indicate that specific cognitive domains may be particularly vulnerable to air pollutant exposure [8,9], especially those associated with executive function and attentional control. Several studies also reported improved cognitive performance under filtered air conditions, highlighting the potential benefits of environmental interventions aimed at improving air filtration [7].

These findings are consistent with broader epidemiological research linking chronic air pollutant exposure to neurodegenerative diseases. The acute effects observed in this review may provide insight into potential immediate physiological mechanisms that contribute to long-term outcomes. Measurable neurobiological stress responses, including a 64% increase in cortisol and elevated vanillylmandelic acid levels following exposure to concentrated coarse particulate matter (PM2.5-10), mirror endocrine profiles observed in high-risk populations [10]. This further supports emerging evidence of biological pathways involving systemic inflammation and stress-mediated neurobiological disruption.

Strengths and Limitations of Included Studies

Included studies had several key strengths, including the use of controlled human exposure designs, which allowed precise manipulation of pollutant concentrations to which participants were exposed [8,9]. Furthermore, validated neuropsychological testing platforms were used consistently across studies to assess cognitive outcomes [7]. However, several limitations should be noted. The number of available clinical trials examining acute air pollution exposure and cognition remains limited, resulting in a relatively small evidence base. Sample sizes across studies were also modest, and participant populations were largely composed of highly educated and relatively affluent individuals. In addition, the controlled nature of pollutant exposure may reduce ecological validity, as real-world exposure typically involves more complex pollutant mixtures and longer exposure durations.

A key limitation of this review is the reliance on a single database (PubMed), which may have restricted the breadth of literature captured and, consequently, the comprehensiveness of the findings. This further highlights the need for additional studies exploring the intersection of acute air pollution exposure and cognitive outcomes in greater detail.

Future Directions

The findings of this review suggest that short-term exposure to air pollutants can result in altered mental states and may produce measurable cognitive and psychological effects in young adults [8-10]. To address this research gap, future studies should move beyond small-scale controlled trials toward longitudinal designs incorporating wearable environmental sensors to capture real-world exposure data. Research should prioritize larger and more diverse participant populations, particularly individuals from lower to middle socioeconomic backgrounds, who are often exposed to higher levels of particulate matter compared with those from higher socioeconomic groups. Interventions aimed at improving air quality may therefore provide benefits that extend beyond respiratory health alone. Longitudinal studies examining chronic exposure to air pollutants will be essential to determine whether acute cognitive effects accumulate over time and contribute to long-term neurological outcomes. Further investigation is also needed to elucidate the underlying biological mechanisms that may link environmental exposures to neurocognitive effects.

## Conclusions

Acute particulate matter exposure may produce measurable changes in cognitive and neurobiological outcomes even in young adults. Evidence from existing studies suggests that pollutants such as particulate matter, diesel exhaust, and elevated carbon dioxide levels can impair multiple cognitive domains. Although the current evidence base remains limited, these preliminary findings highlight the importance of improving air quality and support further research into both the short- and long-term cognitive and neurobiological implications of pollutant exposure.
